# An Effect of a Matrix Made of Cell Wall Polysaccharides from Apple on the Rheological Properties of Various Food Products

**DOI:** 10.3390/polym17182547

**Published:** 2025-09-20

**Authors:** Joanna Mierczyńska, Piotr Mariusz Pieczywek, Justyna Cybulska

**Affiliations:** Institute of Agrophysics, Polish Academy of Sciences, Doświadczalna 4, 20-290 Lublin, Poland; j.mierczynska@ipan.lublin.pl (J.M.); p.pieczywek@ipan.lublin.pl (P.M.P.)

**Keywords:** cell wall polysaccharides, rheology, food thickener, apple pomace

## Abstract

A texture-modifying food matrix (MPS) was obtained by micronizing apple cell polysaccharides and adding spray-dried low-methoxy pectins. This study aimed to demonstrate the effect of MPS addition on a versatile group of products, including instant soup, salad dressing, buttermilk, tomato juice, apple juice, and instant kissel. The rheological properties of suspensions with two MPS concentrations added to these products were compared with those of the control. Additionally, the water holding and retention capacity, swelling capacity, and wetting angles of the MPS and its components were characterized to determine the technological properties of these products. Results show that the MPS proportionally increases viscosity and the thixotropic effect of all studied products, except buttermilk, in relation to concentration. In particular, very pronounced effects were obtained for apple, tomato juice, and salad dressing. All studied suspensions were classified as pseudoplastic fluids; the addition of MPS resulted in varying changes in pseudoplasticity, depending on the product. In summary, this study showed that MPS, as a natural and rich source of dietary fibre matrix, effectively alters rheological properties and may therefore be considered a substitute for other food additives currently used in the food industry.

## 1. Introduction

Nowadays, food additives are commonly used as ingredients, improving many properties of food products. On the one hand, food additives are used to modify foodstuffs and provide convenience in preparation; on the other hand, they are expected to preserve the natural taste, colour, and texture of food. Food texture modifiers are widely studied and described in terms of technological, chemical, and sensory properties, as well as from a rheological perspective [1].

Food additives, according to Codex Alimentarius terminology, are defined as all substances that are generally not consumed separately and are not typically used as ingredients in food products [2]. Consumers usually prefer food products that are free from additives or contain additives of natural origin [3]. Since dietary fibre is of natural origin and has proven health benefits, its chemical and physical properties, such as the ability to swell with water, increase the viscosity of the medium, and form gels, have been intensively studied to improve the quality of food products. Therefore, dietary fibre, also made of by-products or waste material from fruit and vegetable processing, can be used as a food texture modifier [4].

Dietary fibre is composed of plant cell wall polysaccharides, i.e., pectin, cellulose, and hemicelluloses. According to the definition, dietary fibre also consists of lignin, phenolic compounds, waxes, saponins, phytates, cutin, and phytosterols, if they are closely related to cell wall polysaccharides and extracted with them as a fraction of dietary fibre [5]. However, even though these compounds (except lignin) are highly associated with the cell wall, if they are isolated from cell wall polysaccharides, they lose their importance as components of dietary fibre [6]. The technological and physiological properties of dietary fibre depend primarily on its ability to bind water and mechanisms of water holding and retention [7]. The chemical composition, particularly the proportions between soluble and insoluble fractions [8], and microstructural characteristics [7,9] influence the functionality of fibre-based food additives. Dietary fibre-rich products often require rehydration in technological processes; their reconstitution ability is a crucial functional property for agile manufacturing [9]. Soluble dietary fibre fractions facilitate the gelling properties of food products while also enabling optimal gut microbiome balance, possessing prebiotic potential [10]. Insoluble dietary fibre, with its strong hydration properties, has been recognized as beneficial for intestinal health [11]; however, numerous processing methods have been applied to reduce the particle size of this fraction and increase the availability of its components [7,12]. Since dietary fibre has been proven to have health benefits, the challenge and goal are to utilize dietary fibre polysaccharides as a functional food additive [8,13]. Recently, a new food additive made of cell wall polysaccharide matrix from apple pomace (MPS) was developed [14]. A brief description of this technology is presented in the Materials and Methods section. A function of MPS was demonstrated in water solutions with the presence of metal ions. It was shown that MPS has a gelling capability, which is attributed to the micronization of cell wall material and the presence of spray-dried low-methoxyl pectins. Considering that MPS is rich in dietary fibre and would replace currently used texture modifiers, there is a need to test the performance of MPS for changing the rheological properties of real food systems.

This study aimed to demonstrate the extent of changes in the rheological properties of versatile food products, including instant soup, salad dressing, buttermilk, tomato juice, apple juice, and instant kissel, after the addition of MPS.

## 2. Materials and Methods

### 2.1. Preparation of Modified Cell Wall Polysaccharide Matrix (MPS) from Apples

Modified cell wall polysaccharide matrix (MPS) was prepared from apple pomace [14,15]. Pomace from apples purchased from the local market was prepared using a de-pulping machine with a double screw shredder (Twin Gear Juice Extractor, Green Star Elite GSE-5000, Tribest, Anaheim, CA, USA). After drying pomace in a fluidized spray dryer [16] and micronizing it to a particle size of 50–100 µm, the material, designated as S2, was obtained. The prepared S2 material was then stirred with isopropyl alcohol (Stanlab, Lublin, Poland) and homogenized using an ultrasonic processor (VCX-130FSJ, Sonics & Materials Inc., Newtown, CT, USA) for 2 × 30 min, at a frequency of 20 kHz and an amplitude of 57 µm. Suspended S2 was filtered, stirred with 70% ethanol (Stanlab, Lublin, Poland), and mixed with a magnetic stirrer. The sample was stirred with ethanol three times to remove sugars from the S2 material. Desugared S2 was then used for pectin extraction in citric acid (Stanlab, Lublin, Poland). Pectin extraction was conducted in a citric acid solution (pH 2.5) for 30 min at 97 °C, with a ratio of S2 to solution of 1:50. The residues separated during filtration were freeze-dried and labelled as S2L, while the filtrate was concentrated using an evaporator and adjusted to pH 4.5 with aqueous ammonia (Merck Life Science Sp. z o.o., Poznań, Poland). After pectin precipitation with isopropyl alcohol, the obtained polysaccharides were de-esterified with potassium carbonate (Merck Life Science Sp. z o.o., Poznań, Poland) (DM = 37%). Low-esterified pectins were precipitated with ethanol, and after drying at 40 °C, an aqueous solution of apple pectin (AP) was spray dried in a laboratory fluidized spray dryer [16]. The obtained apple pectin spray dried was marked as APS. The two prepared components—S2L and APS—were mixed in the experimentally at an optimized ratio of 2:3 to obtain the final product, polysaccharide matrix (MPS).

### 2.2. Chemical Composition

pH of 1% aqueous suspensions of polysaccharides was measured using a SevenEasy pHmeter with an InLab^®^ Expert Pro electrode (Mettler Toledo, Warsaw, Poland).

The galacturonic acid (GalA) content was determined using a San++ Continuous Flow Analyzer (Skalar, Breda, The Netherlands) according to the colorimetric method [17]. Dried samples were initially hydrolysed using 3M trifluoroacetic acid (TFA, Merck Life Science Sp. z o.o., Poznań, Poland)) solution at 100 °C for one hour, which was then evaporated. Then, the residue was dissolved in ultrapure water produced using a MilliQ system (Merck Millipore, Darmstadt, Germany). Then, samples were injected into the analyzer and totally decomposed in 96% H_2_SO_4_ (Stanlab, Lublin, Poland) containing di-sodium tetraborate (Merck Life Science Sp. z o.o., Poznań, Poland). Then the products were transformed into furfural derivatives, which reacted with the 3-phenyl phenol (Merck Life Science Sp. z o.o., Poznań, Poland) to form a coloured dye, which was measured at 530 nm. Monogalacturonic acid (Merck Life Science Sp. z o.o., Poznań, Poland) solutions were used to create a standard calibration curve.

Cellulose, hemicellulose, pectin, and lignin content were determined according to the van Soest method [18] using a progressive dissolving process in a crude fibre extractor FIWE 3 (Velp Scientifica, Usmate, Italy). First, pectin and other soluble substances such as starch, proteins, fats, and soluble minerals were dissolved in a neutral detergent (sodium dodecyl sulphate, EDTA, pH 7.0, Stanlab, Lublin, Poland). Then, an acid detergent (cetyltrimethyl ammonium bromide (Merck Life Science Sp. z o.o., Poznań, Poland) in 1 N H_2_SO_4_) was applied to dissolve hemicelluloses, and then 72% sulphuric acid was used to dissolve cellulose to determine the lignin content. The content of particular fractions was determined thermogravimetrically.

### 2.3. Water Retention Capacity

Water retention capacity (WRC) is defined as the amount of water bound to the hydrated fibre when an external force is applied [19]. To 1 g of sample, 30 mL of water was added, gently mixed, and hydrated for 20 h, followed by centrifugation (6000× *g*; 20 min) (320 R, Andreas Hettich GmbH & Co. KG, Tuttlingen, Germany). The supernatant was removed by filtering through a nylon membrane (0.20 μm) (Carl Roth GmbH, Karlsruhe, Germany). The weight of the hydrated residue was recorded, and the sample was then dried at 105 °C for 6 h to a dry mass. WRC was calculated according to the following equation:

WRC [g/g] = hydrated sample weight after centrifugation − sample dry weight/sample dry weight

### 2.4. Water Holding Capacity

Water holding capacity (WHC) is defined as the amount of water bound to the fibres without the application of an external force [20]. To 1 g of the sample, 30 mL of water was added, gently mixed, and allowed to hydrate for 20 h. The supernatant was then removed by filtration through a nylon membrane (0.20 μm). The weight of the hydrated sample was recorded, and then the sample was dried at 105 °C for 6 h to obtain a dry mass. WHC was calculated according to the following equation:

WHC [g/g] = hydrated sample weight − sample dry weight/sample dry weight

### 2.5. Swelling Capacity

The swelling capacity (SC) is defined as the ratio of the volume occupied by a sample immersed in excess water after equilibration to its actual weight [20]. To the 0.4 g of sample in the tube, 10 mL of water was added, mixed, and allowed to hydrate for 20 h. Then, the final volume of the sample was measured. SC was calculated according to the following equation:

SC [mL/g] = hydrated sample volume/original sample weight

### 2.6. Measurements of Wetting Angles

**Figure 1:**
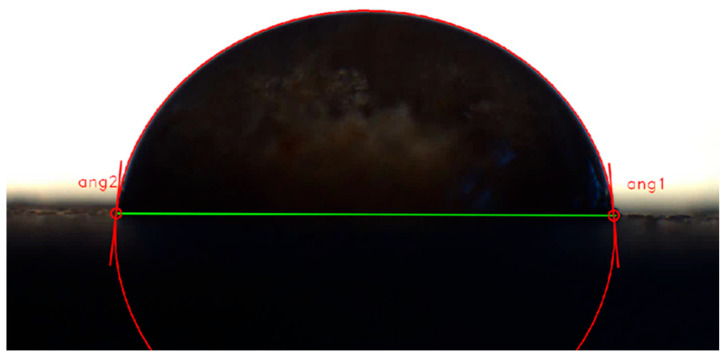
View of a droplet resting on the surface of a polysaccharide matrix as seen in the window of a digital image analysis software.

The wetting angles were measured using an optical goniometer (200-U1, Rame-Hart Instrument Co., Succasunna, NJ, USA). Due to the dynamic nature of the wetting process, an image sequence was recorded for each sample at a frequency of 3.75 frames per second. Images of drops of wetting liquid were recorded using the DFK 51BU02.H CCD camera (Imaging Source Europe GmbH, Bremen, Germany). Deionized water was used as a wetting liquid. The measurements took place at room temperature. Water drops were deposited on the flat surface of the cylindrical samples using a laboratory pipette with a 100 µL capacity (Figure 1). All the droplets were released from a height of 1 cm above the surface.

Measurements of the wetting angle required manual determination of the baseline for each sample. The baseline was determined by selecting the two points at the edge of the drop, where the drop was in contact with the sample surface. Then, using the least squares algorithm, an ellipse was fitted to the edge points of the deposited drop [21]. The wetting angle was calculated as the angle between the baseline and the tangent to the ellipse at the intersection points of the baseline with the ellipse. Left- and right-hand contact angle values were measured separately and then averaged for further analysis.

Wetting angles calculated from the image sequence were labelled with timestamps according to frame number. This allowed the expression of the wetting angle as a function of time. The following exponential function describes the change in the wetting angle in time *θ*(*t*):

(1) $$\theta (t)=\;\theta_{k}+{\alpha e}^{-\beta t}$$

where

*θ_k_*—theoretical, limit value of the wetting angle;
*α*—value by which the wetting angle changes from the initial value to achieve the limit value;
*β*—constant describing the change in the contact angle per unit time;
*t*—wetting time.

The equation was fitted to the values of wetting angles using the least squares algorithm. The term $e^{-\beta }$ describes the relative change in the angle α per unit time. The average time of the decline of angle α can be calculated as $\tau =\frac{1}{\beta }$. Finally, the average velocity of decrease in the wetting angle, expressed in degrees per second, is defined as:

(2) $$\theta =\frac{1}{\tau }$$

In addition, the value of wetting angle (*θ*) after one second from deposition of the water drop was determined from experimental data, as well as the theoretical initial value of the wetting angle defined as:

(3) $$\theta_{t}=\theta_{k}+\alpha$$

**Figure 2:**
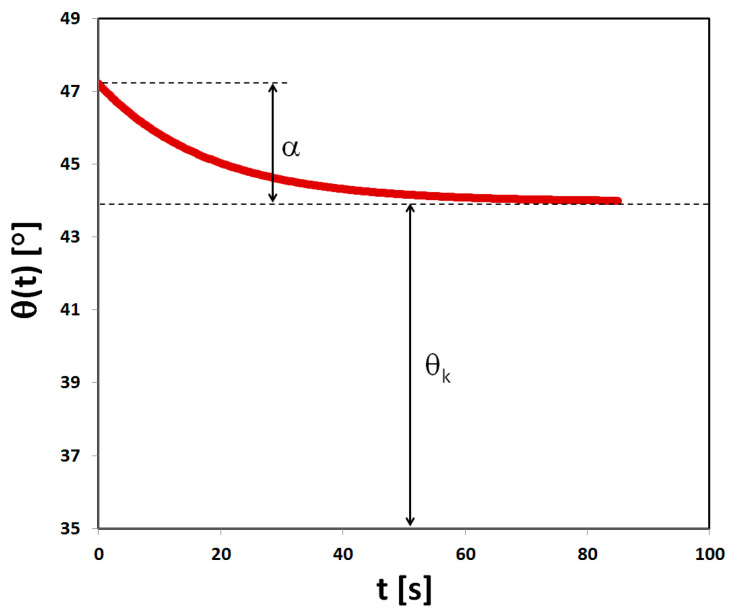
Example plot of the theoretical curve, describing the change in wetting angle over time, with the physical interpretation of the parameters of the Equation (1) plotted.

A theoretical curve describing the change in wetting angle over time is shown in Figure 2.

### 2.7. Preparation of a Suspension of MPS in Food Products

A modified cell wall polysaccharide matrix was used to prepare 2% and 5% suspensions of MPS (mass/volume percentage) in food products, including instant soup, salad dressing, buttermilk, tomato juice, apple juice, and instant kissel. The food products were purchased at a local store and prepared for use according to the manufacturer’s instructions. The nutritional value of the tested food products based on the manufacturers’ declaration is shown in Table 1. Additionally, starch content was determined using the Megazyme total starch assay kit (Megazyme, Wicklow, Ireland). To analyze the properties of MPS in salad dressing, buttermilk, tomato juice, and apple juice, an adequate sample weight was mixed with a sufficient amount of food fluid to obtain 2% and 5% MPS suspensions in a weight ratio. To achieve proper suspensions of MPS in instant soup and instant kissel, the required weight of MPS was mixed with the weight of the instant product, and then the instant food was prepared according to the package instructions. Samples prepared in this way were stored at a temperature of about 2 °C. Before the measurement, the samples were kept at a room temperature of approximately 20 ± 0.5 °C for 1 h.

### 2.8. Determination of Rheological Properties of MPS in Food Product Suspensions

The rheological properties of MPS suspension in food products were measured using an R/S Plus rotational rheometer (Brookfield, Middleboro, MA) with a cone-plate sensor (60 mm diameter, 2° angle) with a 0.5 mm gap between the cone apex and the plate. Measurements were taken at 20 ± 0.5 °C using a constant shear rate (1200 1/s) and a variable shear rate.

The Power law model, also called Ostwald de Waele’s model, was applied to describe the rheological behaviour of flow curves determined experimentally. The model is given as:

(4) $$\sigma =K\cdot \gamma^{n}$$

where σ—shear stress (Pa), K—consistency index (Pas^n^), γ—shear rate (s^−1^), and n—flow behaviour index. Based on the two-parameter rheological model, fluids can be classified as pseudoplastic (shear-thinning) when n < 1 or dilatant (shear-thickening) when n > 1. When the flow behaviour index n = 1, then the consistency index K responds to a constant viscosity.

### 2.9. Statistical Analysis

The data were analyzed using one-way analysis of variance (ANOVA) followed by post hoc tests, and significant differences were determined at *p* < 0.05 using the statistical software STATISTICA (Statistica Version 10, StatSoft Inc., Tulsa, OK, USA).

## 3. Results

The chemical composition of the tested materials is shown in Table 2. Spray-dried apple pectin (APS) contained only minor amounts of hemicellulose. Micronized apple pomace (S2) exhibited a typical chemical composition reported for apple pomace [22,23], which is characterized by approximately 30% of main dietary fibre components such as cellulose, hemicellulose, and pectin [24]. However, proportions between these polysaccharides depend largely on the extraction method and parameters, as well as the apple variety, which can differ significantly [25,26].

Extraction of pectin using citric acid caused a decrease in galacturonic acid content in the S2L sample. Removing pectin also altered the proportions of dietary fibre fractions in this material, with cellulose and hemicellulose fractions becoming the dominant components. The MPS matrix, which is a mixture of APS and S2L materials, was characterized by a high pectin content and significantly reduced cellulose and hemicellulose levels compared to S2. Pectin content determined by the thermogravimetric method was higher than the galacturonic acid content, indicating that, besides galacturonic acid as the main pectin component, other pectin domains were also found in this fraction. Such a complex pectin composition has been previously reported for apple tissue [27,28]. Applying spray-drying to pectin extracted from apple pomace with citric acid improved its solubilization at lower temperatures and reduced the processing time for final food products. Micronization and freeze-drying of apple pomace resulted in obtaining the S2L fraction containing polysaccharide molecules smaller than 50 μm, which is undetectable to consumers. The S2L component also helps stabilize the structure and thicken the food products, while simultaneously providing the insoluble fibre to the food, and providing a way to utilize the residue after pectin extraction.

**Figure 3:**
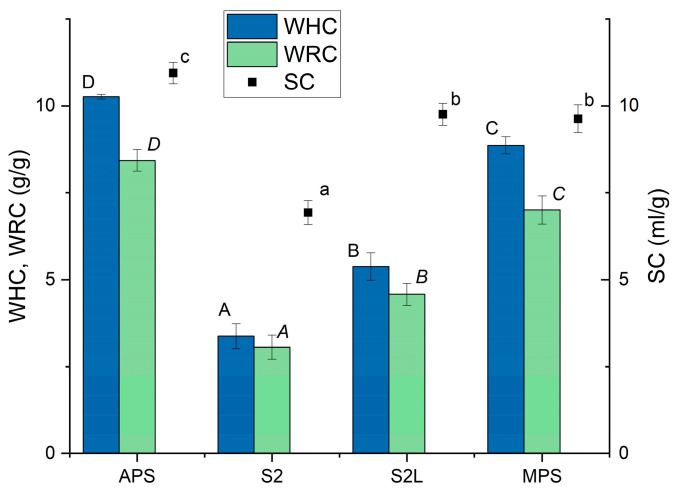
Water holding capacity (WHC), water retention capacity (WRC), and swelling capacity (SC) of spray-dried apple pectin (APS), micronized apple pomace (S2), freeze-dried apple pomace (S2L), and polysaccharide matrix (MPS). Mean values with standard deviations. Values with different letters show statistical significance (*p* < 0.05), a, b, c refer to SC, A, B, C, D refer to WHC, and *A*, *B*, *C*, *D* refer to WRC.

Water holding properties of MPS and its components are presented in Figure 3. Spray-dried apple pectin (APS) was characterized in the highest values of WHC, WRC, and SC. Micronized dried apple pomace with a particle size range of 50–100 μm (S2) had the lowest ability to absorb water, which was evidenced by limited values of WHC and WRC and the lowest SC. WHC and WRC of S2 at the level of approximately 3 g/g (3.37 and 3.06 g/g, respectively) were almost two times higher than reported by Figuerola et al. [29] for dried apple pomace obtained as the by-product in the juice production process, but SC was at the same level. Lyophilization of S2 resulted in the production of the S2L sample, which exhibited significantly increased water holding and retention properties, as well as enhanced swelling capacity. The polysaccharide matrix MPS, a mixture of APS and S2L in a specific proportion, exhibited a swelling capacity equal to that of S2L and comparable to APS. The water holding and water-retention capacities were slightly lower than those in APS, but an apparent synergistic effect of the two components included in the MPS was observed. The water holding capacity reported for pectin varies considerably, ranging from 0.57 g/g [30] to 21.9 g/g [31], depending on the extraction method and source. Developed MPS had comparable water holding and swelling properties to those of apple by-product, obtained through the application of high hydrostatic pressure and a commercial cellulase preparation [32]. High pressure applied to apple pomace, which enabled the accessibility of major soluble polysaccharides, may have had a similar effect to ultrasound applied to the S2 sample, which also caused an increase in water holding properties.

**Figure 4:**
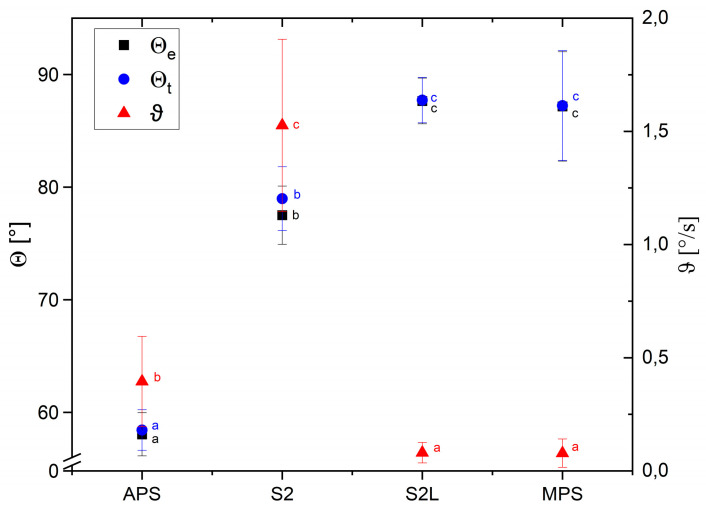
Experimental ($\theta_{e}$) and theoretical ($\theta_{t}$) wetting angle and the velocity of change in the wetting angle in time (θ) of spray-dried apple pectin (APS), micronized apple pomace (S2), freeze-dried apple pomace (S2L), and polysaccharide matrix (MPS). Mean values with standard deviations. Values with different letters show statistical significance (*p* < 0.05).

Due to the dynamic process of wetting, the velocity of change in the wetting angle in time and the theoretical initial value of the wetting angle were determined. The spray-dried pectin sample (APS) was identified as the most hydrophilic material studied, with a wetting angle of approximately 58.4 ± 1.8° (Figure 4). A relatively stable wetting angle value was found for APS compared to S2, which was characterized by the high lability of the wetting angle. Spray-drying of micronized apple pomace, resulting in S2L formation, caused stabilization in the wetting process but simultaneously led to an increase in hydrophobicity. The wetting angles of S2L and MPS reached values of approximately 87° (87.7 ± 2.0° and 87.2 ± 4.8°, respectively), which allows them to be classified as good-wettable materials [33]. S2L and MPS samples exhibited the lowest fluctuation of their contact angles, approaching the final value in the shortest time among the studied samples. Contact angle depends significantly on the particle size of the materials. Calton et al. [9] reported that for fine maltodextrin powder (d50 61.0 ± 5.5 µm), it reached the value of 55.9 ± 4.0°, whereas for maltodextrin coarse (d50 208.0 ± 16.3 µm), the average contact angle was 44.7 ± 3.1°. However, in the case of carrot pomace, the opposite dependence was observed [34]. Carrot pomace particles of a mean size of 279.4 ± 0.8 µm indicated a contact angle of 75.9°, and carrot pomace nanoparticles of a mean size of 203.6 ± 5.1 nm displayed a contact angle of 51.7°. Modifications to the composition of apple pomace-based biocomposites enabled the obtaining of a wide range of contact angle values, from 85.3° to 137.9°, for a superhydrophobic material containing calcium carbonate and beeswax, which can be used as an effective packaging material [35]. The results of our study and literature reports indicate that cell wall polysaccharides have unique potential for tailoring water-binding properties for various applications.

Viscosity and thixotropic effect of food products with the addition of MPS are shown in Table 3. Since the tested food products did not have a high dietary fibre content, the final nutritional value of the food products after the addition of MPS was modified only by the increase in fibre content by 2 or 5%, depending on the MPS dose. The addition of MPS in concentrations of 5% and 2% affected the viscosity of food products in most studied systems, except for the 2% MPS mixture with instant kissel. In most cases, the addition of MPS increased the viscosity of food products. The only exception was the addition of 2% MPS to the buttermilk, where the viscosity decreased compared to the control, but the higher concentration of MPS (5%) added to this product caused a significant increase in its viscosity. The most pronounced effect of MPS addition was obtained in the case of apple juice, which increased from 0.001 Pa·s to 0.020 and 0.214 Pa·s for 2% and 5% MPS, respectively. A similar effect was observed for juice made from tomatoes. The increase in the viscosity of juice products meets consumers’ expectations. Thickening and gelling juices in these cases is a result of a higher concentration of dietary fibre, which is present in food products, and is compatible with consumers’ requests [36]. Clear fruit juices, as shown for apple juice, may have very low viscosity (for clear apple juice, it is close to that of water). Such juices are entirely deprived of dietary fibre, which decreases their nutritional value. Apart from increasing viscosity, the addition of MPS can enrich food products in both soluble and insoluble fractions of dietary fibre, as previously determined for MPS using the Van Soest method [14,18]. MPS contains 32.8% of NDF, which includes 24.2% of ADF (cellulose and lignin) and 8.6% of hemicellulose, and 67.2% of NDS, which is the soluble fibre fraction [14]. The expected increase in viscosity was also observed for the 2% and 5% suspensions of instant soup and salad dressing, as well as the 5% suspensions of buttermilk and instant kissel. Therefore, it can be stated that MPS can be considered a replacement for other thickening agents, such as starch, which is often undesired as a food component due to its unfavourable properties associated with a high post-prandial glycemic response [37].

A hysteresis loop formed between the upward and downward flow curves was used to estimate the thixotropic effect. The values of thixotropic effects for tomato juice, apple juice, salad dressing, and instant kissel suspensions increased with the increase in MPS concentration, which was higher than in the control. For instant soup, 2% MPS caused a decrease in the effect, followed by a pronounced increase over the control sample for 5% MPS added. Only in the case of buttermilk, the addition of MPS caused a slight decrease in the thixotropic effect for both MPS concentrations used. Thixotropy is associated with the complex molecular structure of the analyzed material, based on the electric intermolecular interactions. The addition of MPS in these cases results in the formation of self-organizing systems of particles, caused by the action of weak bonds, such as hydrogen bonds and van der Waals forces [38]. Knowledge of the influence of MPS addition on the thixotropic effect may be helpful in designing food processing systems. Familiarity with thixotropic effect values can help determine the behaviour of food ingredients during stirring, mixing, flow through pipes, and relaxation [39].

**Figure 5:**
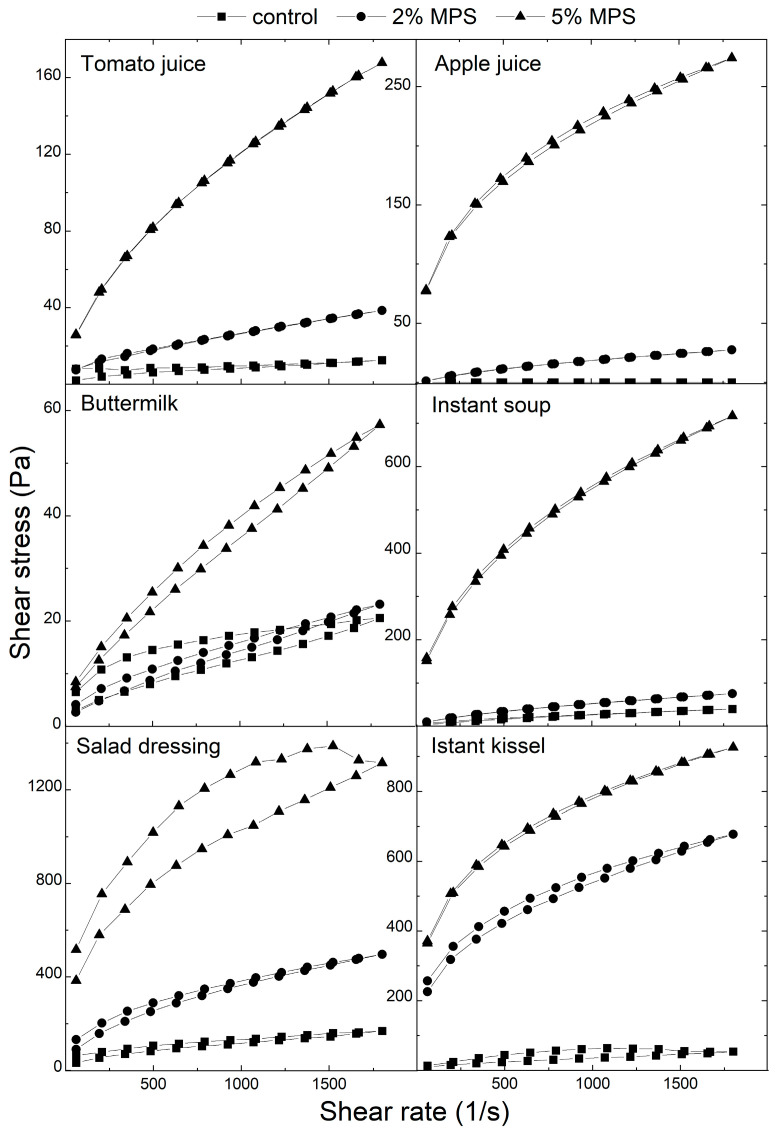
Flow curves (shear stress vs. shear rate) for MPS suspensions in tomato juice, apple juice, buttermilk, instant soup, salad dressing, and instant kissel.

Figure 5 presents flow curves for the analyzed food products. The flow curves were collected using a variable shear rate within the range 60–1800 s^−1^. Both upward and downward curves were fitted with the Power law (Ostwald de Waele’s) model. Parameters of this model are presented in Table 4. Flow curves for instant soup, salad dressing, and instant kissel obtained higher values of shear stress than for tomato juice, apple juice, and buttermilk. The model accurately describes the upward and downward flow curves, as evidenced by an R-squared value of greater than 0.95. Based on the model, all of the samples were classified as pseudoplastic fluids due to n values lower than 1 in each case [40]. The addition of MPS to food products caused different effects on the pseudoplasticity of suspensions. For tomato juice, the addition of MPS caused a decrease in pseudoplastic properties. In the case of apple juice, a decrease in pseudoplasticity was noted for 5% MPS suspensions in apple juice, both for upward and downward flow curves, while it was not observed after the addition of 2% MPS. Buttermilk suspensions with MPS obtained lower pseudoplasticity than in the control for upward curves, and for downward curves with the addition of 5% MPS. Instant soup exhibited higher pseudoplasticity for the 2% MPS suspension and lower pseudoplasticity for the 5% MPS suspension compared to the control, for both upward and downward curves. The opposite properties are presented in the case of upward flow curves of salad dressing—a decrease in pseudoplasticity occurred for 2% suspensions, while an increase was noticed for 5% MPS suspensions. For downward curves, an increase in pseudoplastic behaviour was noticeable in comparison to the control, which presented lower pseudoplasticity than the control upward curve. An increase in pseudoplasticity was observed in the case of instant kissel, accompanied by a decrease in n values for both upward and downward flow curves.

The consistency index K is connected with the viscosity of samples; higher values of the consistency index can be equivalent to an increase in viscosity. Generally, the addition of MPS resulted in an increase in K index values for most products. This tendency was not only present in the case of upward curves of MPS suspensions in buttermilk.

Analysis of the rheological properties of the selected food products enriched with the MPS addition revealed a beneficial influence of this polysaccharide matrix on technological properties, especially in apple and tomato juices, salad dressing, and, at higher concentrations of the MPS, instant soup and kissel. The ability to bind water molecules and thereby control the viscosity of buttermilk with the MPS addition was moderate. The pronounced effect of the MPS matrix in food products containing starch (instant soup, salad dressing, kissel) may result from synergistic interactions between pectin and starch [41]. Conversely, pectins have been reported as ineffective in stabilizing high-protein milk beverages [42], which explains their limited influence on the rheological properties of buttermilk. However, the results indicate that in some applications, the MPS matrix can be considered as a substitute for popular thickeners or stabilizers such as starch, arabic gum, sodium alginate, or xanthan, which have mild structurizing properties [4]. Additionally, as some common food stabilizers are being re-evaluated as food additives due to concerns about their impact on human health, pectin and apple dietary fibre, which have proven health benefits, can be an alternative and safe solution for the food industry and consumers [43,44,45,46,47].

## 4. Conclusions

The presented study demonstrated that the polysaccharide matrix MPS, composed of natural dietary fibre from apples, may serve as an effective food texture modifier. Applied processing of apple dietary fibre polysaccharides resulted in enhancing water binding properties and stability of the wetting angle. All the components of the MPS matrix exhibited a hydrophilic character, which enabled the structurization of food products. The addition of MPS polysaccharide matrix to analyzed products, including instant soup, salad dressing, tomato juice, apple juice, and instant kissel, caused changes in the rheological properties of the food fluids; however, the extent of these changes varied. An exception was the different behaviour after the addition of MPS to buttermilk. Generally, the addition of MPS in concentrations of 2% and 5% resulted in an increase in both the thixotropic effect and viscosity. Ostwald de Waele’s model, fitted to flow curves, shows that the addition of MPS affects the pseudoplasticity of the samples. This experiment has demonstrated that the polysaccharide matrix MPS, based on natural dietary fibre from apples, can be used as a food texture modifier of natural origin with the capability to replace commonly used commercial food additives.

## Figures and Tables

**Table 1:** Nutritional value of the studied food products.

	Tomato Juice	Apple Juice	Buttermilk	Instant Soup *	Salad Dressing	Instant Kissel *
Energy (kJ/kcal)	77/18	189/45	178/42	92/22	727/179	223/52
Protein (g)	0.8	<0.5	3.5	1.0	0.8	0.0
Carbohydrate (g)	2.7	11.0	4.8	3.8	8.0	13.0
of which sugars (g)	2.7	11.0	4.0	1.2	6.0	7.7
of which starch (g)	0.1	nd	nd	0.7	1.9	5.1
Fat (g)	<0.5	<0.5	1.0	0.2	16.0	0.0
of which saturates (g)	<0.1	0.0	0.6	0.1	1.2	0.0
Fibre (g)	0.5	nd	nd	0.1	nd	nd
Salt (g)	0.70	0.00	0.08	0.66	2.30	0.01

* in 100 mL of prepared product; nd—not determined.

**Table 2:** Chemical composition of spray-dried apple pectin (APS), micronized apple pomace (S2), freeze-dried apple pomace (S2L), and polysaccharide matrix (MPS).

	APS	S2	S2L	MPS
pH	3.26 ± 0.01	3.90 ± 0.01	4.21 ± 0.01	3.4 ± 0.0
Galacturonic acid content (mg/g)	694.1 ± 1.0	172.5 ± 5.6	82.5 ± 3.1	389.8 ± 5.6
Dietary fibre fractions (%)				
Cellulose	0.0 ± 0.0	37.8 ± 0.7	48.8 ± 0.7	20.8 ± 0.6
Hemicellulose	1.1 ± 0.1	26.1 ± 0.5	33.4 ± 0.5	10.5 ± 0.7
Pectin *	98.9 ± 0.1	30.8 ± 0.4	9.0 ± 0.4	65.4 ± 0.3
Lignin	0.0 ± 0.0	5.3 ± 0.4	8.8 ± 0.3	3.3 ± 0.1

* Pectin fraction can contain other soluble solids such as protein, fat, starch, and mineral substances.

**Table 3:** Viscosity (Pa·s) and thixotropic effect (kPa·s^−1^) for suspensions of MPS in food products. Mean values with standard deviations. Values with different letters show statistical significance (*p* < 0.05).

		Tomato Juice	Apple Juice	Buttermilk	Instant Soup	Salad Dressing	Instant Kissel
Viscosity (Pa·s)	Control	0.011 ^a^ ± 0.001	0.001 ^a^ ± 0.001	0.020 ^a^ ± 0.001	0.035 ^a^ ± 0.009	0.089 ^a^ ± 0.013	0.530 ^a^ ± 0.091
	2% MPS	0.025 ^b^ ± 0.004	0.020 ^b^ ± 0.001	0.014 ^b^ ± 0.001	0.014 ^b^ ± 0.001	0.327 ^b^ ± 0.052	0.530 ^a^ ± 0.086
	5% MPS	0.128 ^c^ ± 0.020	0.214 ^c^ ± 0.016	0.038 ^c^ ± 0.012	0.519 ^c^ ± 0.137	0.671 ^c^ ± 0.041	0.718 ^b^ ± 0.096
Thixotropic effect (kPa·s^−1^)	Control	0.300 ^a^ ± 0.137	nd	8.640 ^a^ ± 2.973	7.726 ^a^ ± 1.554	43.228 ^a^ ± 6.861	69.532 ^a^ ± 4.526
	2% MPS	5.742 ^b^ ± 0.991	3.254 ^b^ ± 0.951	5.197 ^b^ ± 1.322	3.542 ^b^ ± 0.344	92.837 ^b^ ± 32.594	134.215 ^b^ ± 10.333
	5% MPS	21.221 ^c^ ± 6.666	28.055 ^c^ ± 5.758	7.574 ^c^ ± 3.142	76.235 ^c^ ± 5.211	570.483 ^c^ ±192.995	115.458 ^c^ ± 19.591

nd—not determined.

**Table 4:** Parameters for the Power Law model describing rheological properties of MPS suspensions in food products. K—consistency index (Pas^n^); n—flow behaviour index; R^2^—determination coefficient.

Food Product	Sample	Upward Curve			Downward Curve		
		K (Pas^n^)	n	R^2^	K (Pas^n^)	n	R^2^
Tomato juice	Control	1.00 ± 0.00	0.30 ± 0.01	0.99	2.76 ± 1.52	0.20 ± 0.01	0.99
	2% MPS	1.06 ± 0.01	0.47 ± 0.04	0.99	1.00 ± 0.00	0.47 ± 0.03	0.99
	5% MPS	2.11 ± 0.61	0.57 ± 0.06	0.99	2.077 ± 0.74	0.57 ± 0.01	0.99
Apple juice	Control	nd	nd	nd	nd	nd	nd
	2% MPS	1.08 ± 0.06	0.45 ± 0.03	0.98	1.00 ± 0.00	0.43 ± 0.01	0.98
	5% MPS	14.82 ± 5.61	0.38 ± 0.02	0.96	14.86 ± 6.21	0.38 ± 0.03	0.95
Buttermilk	Control	2.64 ± 1.42	0.29 ± 0.11	0.99	1.00 ± 0.01	0.37 ± 0.02	0.99
	2% MPS	1.00 ± 0.00	0.40 ± 0.02	0.99	1.005 ± 0.00	0.37 ± 0.02	0.99
	5% MPS	1.02 ± 0.03	0.52 ± 0.06	0.99	1.007 ± 0.00	0.51 ± 0.05	0.99
Instant soup	Control	1.29 ± 0.05	0.46 ± 0.05	0.99	1.002 ± 0.02	0.47 ± 0.01	0.99
	2% MPS	1.74 ± 1.25	0.39 ± 0.02	0.99	1.91 ± 1.58	0.44 ± 0.09	0.99
	5% MPS	16.05 ± 2.72	0.48 ± 0.02	0.99	15.73 ± 2.49	0.49 ± 0.02	0.99
Salad dressing	Control	16.41 ± 6.23	0.32 ± 0.06	0.99	3.32 ± 0.85	0.53 ± 0.01	0.99
	2% MPS	22.54 ± 10.88	0.41 ± 0.07	0.99	10.21 ± 1.54	0.51 ± 0.01	0.99
	5% MPS	186.97 ± 82.02	0.28 ± 0.05	0.99	83.20 ± 29.87	0.36 ± 0.03	0.99
Instant kissel	Control	6.53 ± 1.12	0.58 ± 0.03	0.99	8.21 ± 0.03	0.55 ± 0.01	0.99
	2% MPS	67.16 ± 7.69	0.29 ± 0.00	0.99	47.47 ± 6.21	0.34 ± 0.00	0.99
	5% MPS	107.58 ± 8.05	0.27 ± 0.01	0.99	110.21 ± 9.30	0.27 ± 0.01	0.99

nd—not determined.

## Data Availability

The original contributions presented in this study are included in the article. Further inquiries can be directed to the corresponding author.
